# Supplementary figures and images for: Soil Geochemical Properties Influencing the Diversity of Bacteria and Archaea in Soils of the Kitezh Lake Area, Antarctica
Source: Biology (Basel). 2022 Dec 19;11(12):1855. doi: 10.3390/biology11121855 (PMC9775965; doi:10.3390/biology11121855)

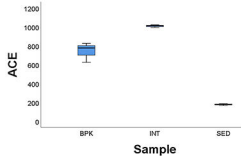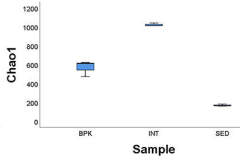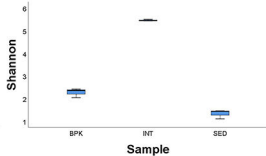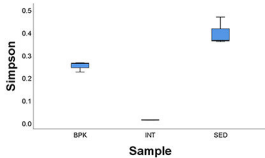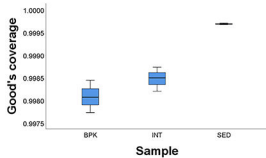

Supplement: Supplementary file 1 [file biology-11-01855-s001.zip › Figure S1a.pdf]

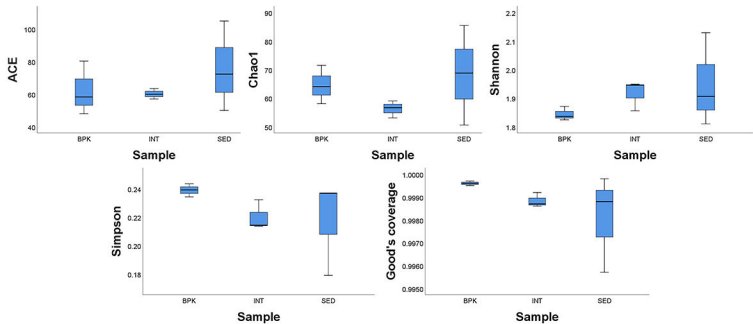

Supplement: Supplementary file 1 [file biology-11-01855-s001.zip › Figure S1b.pdf]

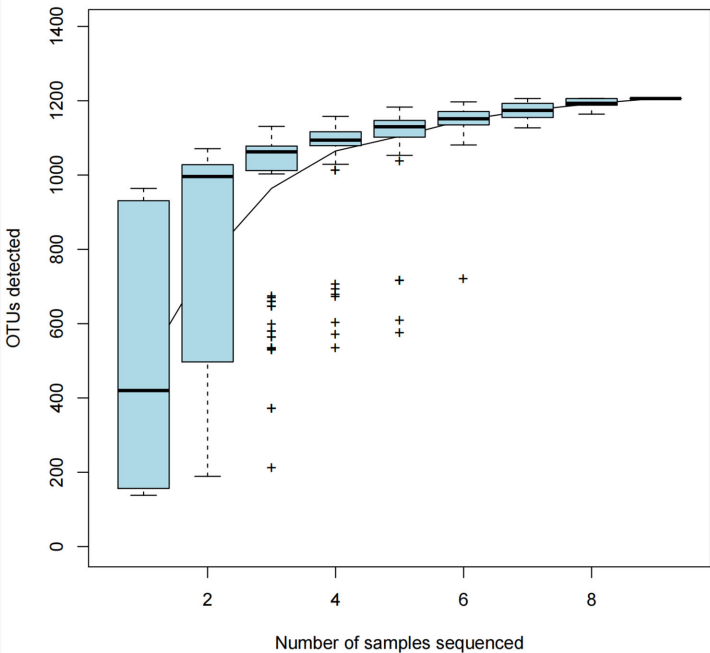

Supplement: Supplementary file 1 [file biology-11-01855-s001.zip › Figure S2a.pdf]

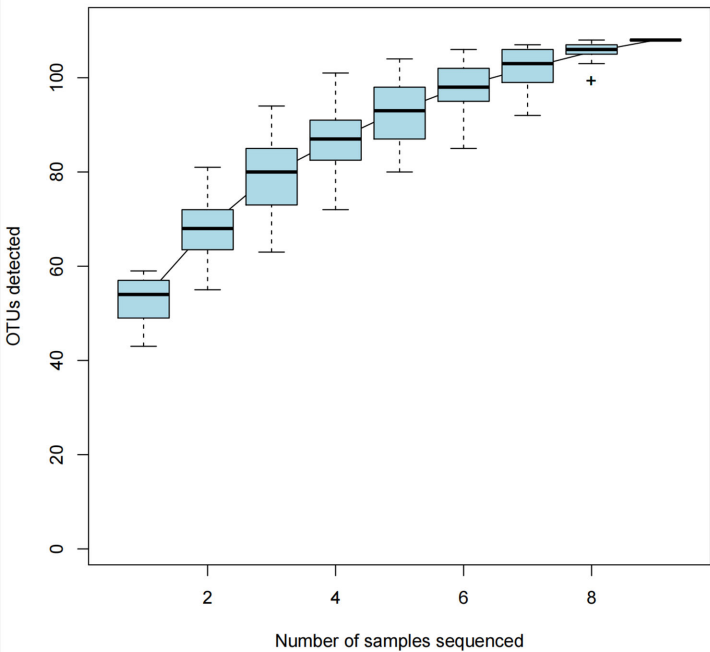

Supplement: Supplementary file 1 [file biology-11-01855-s001.zip › Figure S2b.pdf]
